# A systematic review exploring youth peer support for young people with mental health problems

**DOI:** 10.1007/s00787-022-02120-5

**Published:** 2022-12-10

**Authors:** C. R. M. de Beer, L. A. Nooteboom, L. van Domburgh, M. de Vreugd, J. W. Schoones, R. R. J. M. Vermeiren

**Affiliations:** 1 LUMC Curium, Child and Adolescent Psychiatry, Leiden University Medical Center, Leiden, The Netherlands; 2 Department of Child and Adolescent Psychiatry and Psychosocial Care, Amsterdam University Medical Center, Amsterdam, The Netherlands; 3 iHUB, Rotterdam, The Netherlands; 4 Directorate of Research Policy (Formerly: Walaeus Library), Leiden University Medical Center, Leiden, The Netherlands; 5 Youz, Parnassia Psychiatric Institute, The Hague, The Netherlands

**Keywords:** Youth peer support workers, Youth peer support, Mental health problems, Adolescents, Young adults, Child and adolescent mental health services

## Abstract

Youth peer support workers (YPSWs) are young adults with lived experience of mental illness during childhood or adolescence who support young people receiving treatment in mental health services. The contributions made by YPSWs are a promising development to facilitate consumer-centered and recovery-oriented care. Although the youth peer support workforce is expanding rapidly, structurally embedding YPSWs in practice is challenging. To overcome these challenges and thereby improve care for young people, insight into YPSW roles, barriers and facilitators for implementing and pursuing youth peer support (YPS) is a necessity. This systematic review examined the published literature to identify existing knowledge on YPSW roles in treatment settings, and the barriers and facilitators for implementing and pursuing YPS in practice. A total of 24 studies from a variety of youth serving contexts were included in this review. Thematic synthesis resulted in six YPSW roles and five themes with barriers and facilitators. The roles included the: engagement role, emotional support role, navigating and planning role, advocacy role, research role and the educational role. The themes explored the needs of YPSWs, experiences of YPSWs, relationships between service users and YPSWs, the collaboration process between YPSWs and non-peer staff, and organizational readiness. This review underlines that YPSWs likely are a valuable addition to numerous youth treatment contexts. Overall, the implementation of YPSWs is a multifaceted operation that requires careful planning. We recommend services to set clear and realistic expectations for YPSWs, to consider potential power imbalances between YPSWs and non-peer staff, to provide adequate resources to pursue YPS, and to approach the implementation of YPSWs with a growth mindset.

**Supplementary Information:**

The online version contains supplementary material available at 10.1007/s00787-022-02120-5.

## Introduction

When severe mental illness strikes during childhood or adolescence, it can have lifelong adverse consequences. Consequences include destructive coping skills, low self-esteem, increased economic burden, poor psychosocial functioning, and mental illness later in life [1–3]. Youth mental health services provide various treatment programs to support young people with severe mental illness [4]. However, many young people find that the existing treatment programs do not fully meet their needs [1, 4–6]. To meet the needs of these young people, it is necessary that services put more emphasis on recovery, consumer-centered care and empowerment of service users, and less emphasis on traditional clinical models focusing on treating psychiatric symptomatology [6–8]. A promising approach to assist youth mental health services in providing more recovery-oriented care is the involvement of peer support workers (PSWs) [9, 10].

In the last 2 decades, peer support work has become increasingly popular in mental health programs across westernized countries [11]. A PSW is identified as someone with lived experience of mental illness who supports others in recovery from mental illness [11, 12]. As demonstrated by multiple studies, involving PSWs in mental health services promotes recovery and can have far-reaching positive impacts [8, 13, 14]. These positive impacts include increased hope, empowerment, self-esteem, and increased treatment engagement of service users [14]. While these results are promising, these studies mainly concern adult mental health services [10, 15].

A growing number of studies is exploring the involvement of PSWs in youth mental health services [9, 10, 15–18]. These PSWs are typically referred to as youth peer support workers (YPSWs). Studies suggest that YPSWs promote treatment engagement and enable young people to better manage family and community stigma regarding mental illness [17, 18]. Moreover, studies also suggest young people perceive YPSWs as more reliable compared to non-peer staff, because YPSWs self-disclose lived experience of mental illness and hardships [16]. Even though these studies underline the need for YPSWs in youth mental health services, most of these studies are small-scale program evaluations or studies to assess YPSW interventions for young people with a single disorder [9, 18]. An overview of the existing knowledge on YPSWs in youth mental health services is lacking. Such an overview can facilitate the involvement of YPSWs in practice as it can shed light onto the diverse YPSW roles, and barriers and facilitators for implementing and pursuing youth peer support programs.

Thus, to guide the involvement of YPSWs in practice and consequently improve mental health programs for young people with mental illness, an overview of the existing literature and knowledge gaps on YPSWs is required. Therefore, this study aims to systematically review current literature to identify what we know so far about the YPSW roles in treatment settings, and the barriers and facilitators for implementing and pursuing youth peer support programs in practice. Insight into these roles, barriers and facilitators will result in practical recommendations for embedding YPSWs in youth mental health services.

## Methods

A research protocol for this systematic review was developed in accordance with the Preferred Reporting Items for Systematic Reviews and Meta-Analysis (PRISMA) guidelines [19]. This research protocol was registered in the International Prospective Register of Systematic Reviews (PROSPERO: registration number CRD42021236588).

### Including a youth peer support worker on the review team

This systematic review was conducted in collaboration with a YPSW (MV) on the review team. The YPSW is specialized in research and has experience in delivering peer support to young people aged 12-18 inpatient at Curium, the department of child and adolescent psychiatry of the Leiden University Medical Center. The YPSW was actively involved during the screening stage of this systematic review and had an advisory role during the data synthesis process.

### Search strategy

The search strategy was designed in collaboration with an experienced information specialist (JS). Studies published between January 2000 and June 2022 and meeting the inclusion criteria were screened. The following electronic databases were searched: PsycINFO (EBSCOhost), Embase (OVID), MEDLINE (OVID), Cochrane Library, Web of Science, PubMed, Emcare (OVID), Academic Search Premier (EBSCOhost), Social Services Abstracts (ProQuest), and Sociological Abstracts (ProQuest). The search strategy included a combination of controlled vocabulary words and free text words related to YPSWs, child and adolescent mental health services, and mental health problems within children and adolescents. After running a preliminary search, the search strategy was further adapted by including additional terms found in published articles on youth peer support [16, 17, 20]. The complete search strategy can be found in appendix A. To avoid missing relevant publications, the reference lists of studies selected for data extraction were screened for additional studies. All identified studies were recorded in the reference management software EndNote^®^.

### Eligibility criteria

**Fig. 1:**
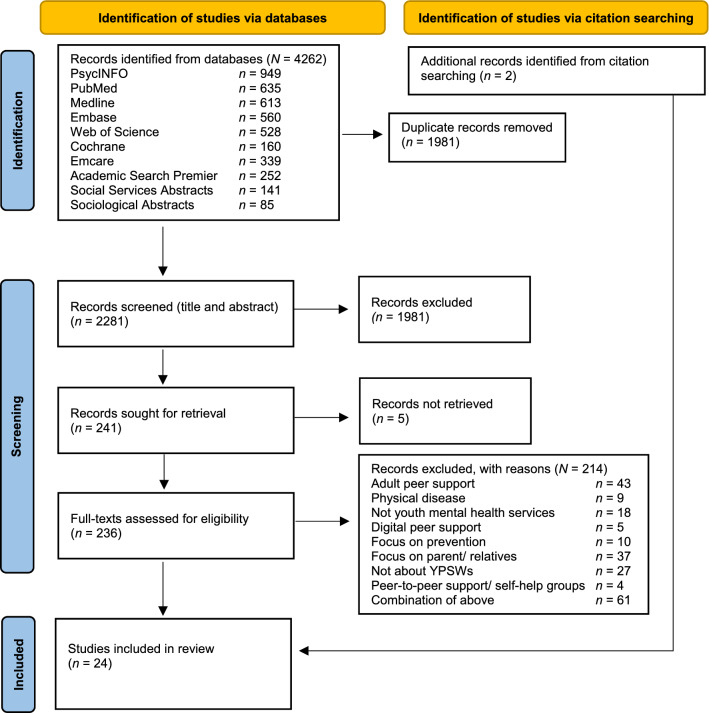
PRISMA flow chart

Both screening rounds (title and abstract screening in round one, and full text screening in round two) were done by two independent authors (CB and MV), using predefined inclusion and exclusion criteria. The process was recorded by the PRISMA flow diagram (see Fig. 1). To be included, studies had to meet the following criteria:

- Participant and intervention: studies on YPSWs supporting people aged 8–26 in youth mental health services. In this study youth mental health services referred to services ranging from primary care community services to specialist care mental health services [1]. This study defined mental health problems as any behavioral, emotional, or developmental problem causing mild to severe impairment [21].
- Outcome: study outcomes reporting on YPSW roles (i.e., responsibilities, tasks and characteristics of YPSWs), and/or barriers (i.e., aspects that prevented or had negative outcomes on peer support work) and/or facilitators (i.e., aspects that improved, enabled, enhanced peer support work) of pursuing or integrating youth peer support workers.
- Study design: all study designs (i.e., qualitative, quantitative, and mixed-method) were included in this systematic review.
- Language and year: publications were included if they were peer reviewed, reported in English or Dutch, and were published between January 2000 and June 10, 2022. Since our aim was to provide an overview of methodologically rigorous information on youth peer support, we did not include grey literature [16].

To ensure we collect as much information as possible on the characteristics of YPSWs, we included no age range for YPSWs in our eligibility criteria. Specific exclusion criteria were: studies focusing on PSWs in adult mental health services, YPSWs for the prevention of mental illness, YPSWs for parents and/or relatives, YPSWs for young people with a physical disease, and YPSWs in digital environments. However, digital interventions embedded in in-person services were included.

### Data extraction and synthesis

After study selection took place, the first author (CB) carefully extracted the data under supervision of a qualified qualitative review author (LN). We used a pre-designed data extraction table with pre-designed data fields. The data extraction fields included the following study details: title, publication type, authors, date, study description and context, study methodology, sample demographics, interventions, and study quality appraisal. Moreover, the following study outcomes were recorded based on our research questions: (1) the context and types of youth peer support; (2) the key features of YPSW roles; (3) the facilitators and barriers for implementing and pursuing peer support.

After data extraction took place, we started the thematic synthesis with the open coding of the key features of YPSW roles, and facilitators and barriers for implementing and pursuing youth peer support in practice [22]. This approach consisted of three steps and was conducted by the first author (CB) under supervision of another review author (LN). First, line-by-line coding of the included full text articles took place. Subsequently, descriptive themes were developed by grouping together similar codes from the first step and renaming them with an overarching code. Finally, the author (CB) went beyond the descriptive themes to generate subthemes to answer the review questions of this systematic literature review [23]. These overarching themes and subthemes were discussed multiple times in the review team to reach consensus.

### Quality appraisal

The quality of the publications was assessed using the Critical Appraisal Skills Programme (CASP) 2018 checklists. Since the CASP checklists do not provide an overall score, the scoring system used in a study by Ibrahim et al. (2020) was applied [24]. One point was given to each item that was rated with ‘yes’. Items rated ‘no’ were given 0 points. Each CASP checklist consisted of 10 to 12 items. After completion, a percentage score was calculated for each checklist to grade the study quality. Studies scoring ≥ 60% were graded as good quality, studies scoring 45–59% were graded as fair, and studies scoring < 45% were graded as poor [24].

### Strength of evidence

After completing data extraction, quality appraisal and thematic synthesis, the strength of evidence of the formulated themes was assessed. The strength of evidence was calculated for each subtheme based on the quality of studies, size of evidence, context, and consistency [25–27].

- Quality of studies: based on the critical appraisal checklists for individual studies the overall quality of the subtheme was assessed. Good (+): was awarded to subthemes made up of > 75% of studies appraised as high quality. Fair (±): was awarded to subthemes made up of 25–75% of high-quality studies. Poor (-): was awarded to subthemes made up of < 25% high-quality studies.
- Size of evidence: the size of evidence was calculated using the number of studies within a subtheme. Subthemes consisting of 10 or more studies were graded as large (+), subthemes made up of 5 to 9 studies were graded as medium (±), and subthemes made up of 4 or less studies were graded as small (-).
- Generalizability: the context of each subtheme was categorized into global or specific. General (+) was assigned to subthemes consisting of studies from a variety of contexts, and specific (-) was assigned to subthemes made up of studies within the same specific context.
- Consistency: Subthemes made up of evidence pointing to similar conclusions were considered consistent (+), subthemes made up of mixed conclusions from multiple studies from different contexts were considered mixed (±), and subthemes made up of one or more studies from the same context that refutes the conclusions from other studies within the same context were graded as inconsistent (-).

Based on the scores assigned in each subscale (quality of studies, size of evidence, generalizability and consistency), the overall strength of evidence was calculated: very strong (++++), strong (+++), medium (++), weak (+), or no evidence (−).

## Results

### Study characteristics

A total of 24 studies were included for analysis in this systematic review. Of the 24 studies, all studies were published within the last 8 years (2014–2022). The included studies covered multiple settings, including: child and adolescent psychiatry services (*n* = 12), community services for youth with mental health problems (*n* = 5), youth offending services (*n* = 3), community services for homeless youth (*n* = 3), and a scoping review that discussed a variety of youth serving contexts (*n* = 1). Most of the included studies focused on youth peer support in outpatient settings (*n* = 20). A total of 23 studies reported on how YPSWs delivered peer support. In 4 studies, YPSWs delivered group peer support, in 5 studies, YPSWs delivered individual peer support, in 12 studies, YPSWs delivered both individual and group peer support, and in 2 studies, YPSWs provided lived experience insight to the organization and teams. In terms of age, most studies (*n* = 17) focused on youth peer support for young people aged 13 to 26. A total of 16 studies included details on the age range of the YPSWs. Of these studies, 6 reported on YPSWs in the same age range as the service users, 5 reported on YPSWs a few years older than the service users, and 5 studies reported on YPSWs of all ages. In the majority of studies, YPSWs were the primary respondents (*n* = 15). A total of 12 studies reported on YPSW supervision, 7 studies mentioned YPSWs received supervision from staff and 5 studies reported YPSWs received supervision both from staff and groups led by a peer supervisor.

The study methodology ranged across studies, 19 studies were descriptive, and 5 studies were analytic. The quality of the individual studies was assessed using the CASP checklists. We assessed 20 studies as high quality, and 3 studies were assessed as fair quality. One study was assessed as poor quality because it lacked a clear report on the methodology of data collection. In Appendix B an overview of the study characteristics can be found.

### Outcomes

Axial coding resulted in a total of six roles for YPSWs in practice. See Table 1 for an overview of YPSW roles in youth mental health services and their associated strength of evidence. The coded barriers and facilitators were grouped together in overarching themes as they were often directly linked to one another. An overview of the themes and subthemes can be found in Table 2.

**Table 1:** Overview of the strength of evidence and roles YPSWs in treatment settings

Roles youth peer support workers	Number of studies	Quality of studies	Context	Consistency	Overall strength of evidence
Engagement role	21	Good	General	Consistent	Very strong
Emotional support role	17	Good	General	Consistent	Very strong
Navigating and planning role	11	Good	General	Consistent	Very strong
Advocacy role	10	Good	General	Consistent	Very strong
Research role	3	Fair	General	Consistent	Medium/strong
Educational role	6	Good	General	Mixed	Strong

**Table 2:** Themes and subthemes for the barriers and facilitators in the implementation and pursuit of youth peer support services

Theme	Subtheme	Number of studies	Quality of studies	Context	Consistency	Strength of evidence
Needs of YPSWs	Supervision	12	Good	General	Consistent	Very strong
	Training and education	10	Fair	General	Consistent	Strong/very strong
	Flexibility	6	Fair	General	Consistent	Strong
Experiences of YPSWs	Identity transition	11	Good	General	Consistent	Very strong
	Control	2	Good	Specific	Consistent	Medium
	Personal factors associated with job success of YPSWs	7	Good	General	Consistent	Strong/very strong
Relationship young service users and YPSWs	Boundaries	10	Good	General	Consistent	Very strong
	Non-judgmental attitude	7	Good	General	Consistent	Strong/very strong
Collaboration YPSWs and non-peer staff	Concerns and attitudes	9	Good	General	Consistent	Strong/very strong
	Co-production	8	Good	General	Consistent	Strong/ very strong
	Role clarity	12	Good	General	Mixed	Strong/very strong
Organizational readiness	Organizational requirements	14	Fair	General	Mixed	Strong
	Training non-peer staff	4	Good	General	Consistent	Strong
	Added value of YPSWs for the organization as a whole	10	Fair	General	Consistent	Strong/very strong

The section below first describes the roles for YPSWs in practice. Following the roles, the barriers and facilitators implementing YPSWs in practice will be described. To see a detailed overview of the study characteristics for the included studies please see appendix B. An overview of all themes, subthemes and the calculation of the strength of evidence can be found in appendix C.

### Roles youth peer support workers in practice

The six roles we identified included: the engagement role, the emotional support role, the navigating and planning role, the advocacy role, the research role, and the educational role. Table 1 presents an overview of the strength of evidence for YPSW roles in treatment settings. A high score on the strength of evidence (strong to very strong) means that numerous high-quality studies in a variety of contexts point to similar results. In this section, we provide a detailed description of the six roles we identified in the literature.

### Engagement role

The engagement role of YPSWs was reported in most of the included studies, and involves building trust, reaching out, reducing isolation, and (re-) engaging young persons in youth mental healthcare [9, 10, 16, 18, 20, 28, 28–37]. YPSWs are uniquely qualified for this role as their lived experience enables YPSWs to start relationships grounded on equality, authentic empathy and a non-judgmental attitude [17, 20, 28–31, 36, 39, 40]. One study suggested that by sharing lived experience in response to topics discussed in group settings, YPSWs facilitate mutual sharing, which reduces isolation by reframing group participants’ perception of struggling alone with mental health challenges [34]. Moreover, YPSWs are often close in age to these young persons’ using services. This allows YPSWs to engage young persons by offering developmentally and culturally appropriate support [4, 16, 17, 20, 28, 29, 31, 33, 39, 40]. This deeper understanding of youth culture also allows YPSWs in the engagement role to assist services in changing the atmosphere to become more welcoming for young people [9, 20, 41]. For example, one study described YPSWs can transform the atmosphere of waiting rooms through comforting and talking to young service users by sitting with them while they wait for appointments [20]. Furthermore, studies underlined the involvement of numerous YPSWs within services facilitates engagement of youth; it allows young persons to be matched to YPSWs based on ethnicity, background, and a variety of personal experiences [17, 33].

### Emotional support

Within treatment settings, YPSWs can provide emotional support to young persons. In this role, a variety of studies reported that YPSWs empower, comfort, provide hope, sympathy, build trust, affirm, and manage expectation of young service users [9, 10, 15, 16, 18, 20, 28–31, 34–37, 40, 42, 43]. By virtue of their lived experience, YPSWs have a deeper understanding of the issues presented by young service users. This deeper understanding enables YPSWs to validate feelings associated with adverse experiences, and to provide emphatic confrontation for current maladaptive behaviors [36, 40]. Moreover, through modeling recovery, YPSWs can stimulate self-acceptance, positive identity formation, and give young person’s perspective by providing a real-life example that recovery is attainable [16, 31, 36, 40, 42].

### Navigating and planning role

Within the navigating and planning role, multiple studies described that YPSWs can support service users and treatment teams in prioritizing and planning treatment based on the personal goals of the young person [16, 18, 20, 31, 33, 36, 38, 41, 42]. Moreover, many YPSWs have a wide range of experiences within the mental health care system. YPSWs can use these experiences to help young persons, staff and caretakers in navigating and finding appropriate mental health services [10, 15, 31, 42]. For example, one study reported YPSWs can facilitate the transition from youth to adult mental health services by joining service users to intakes in adult services [10].

### Advocacy role

YPSWs commonly have a large role in advocacy activities. Advocacy activities include: attending treatment team consultations, training staff, helping design policies within youth services, bridging service users and non-peer staff, and sharing stories to reduce stigma and discrimination towards people with mental illness [10, 15, 16, 18, 20, 33, 34, 36, 37, 39]. YPSWs in the advocacy role can have collective benefits for the mental health service as a whole, including a culture change towards more recovery-oriented care [20, 37].

### Research role

Another role for YPSWs is the research role. Three studies described when designing new programs, interventions, and evaluating services, having YPSWs in the role of researcher can give an “insider” perspective in the data analysis and design process. This ensures the program and interventions are relevant for the target population and deepens the understanding of the results [16, 18, 30].

### Educational role

Finally, some studies described YPSWs can utilize lived experience to educate young persons, their friends and family about mental challenges in the educational role [16]. Since YPSWs are realistic role models, they can set an example for finding meaning and moving on after having lived through mental distress. In doing so, YPSWs model skills, attitudes and behaviors to young service users [28, 31, 36]. Besides, YPSWs can educate healthcare professionals by sharing experiential expertise and providing insight in the recovery process [15, 18]. Education provided from a lived experience perspective to healthcare professionals can prompt a shift from a traditional clinical deficit model to recovery-focused care [15].

### Barriers and facilitators

The barriers and facilitators for implementing and pursuing youth peer support were grouped together through overarching themes and subthemes. The thematic clustering resulted in 5 overarching themes and 14 subthemes. See Table 2 for an overview of the strength of evidence for each subtheme.

### Theme 1: *needs of youth peer support workers*

The theme ‘needs of youth peer support workers’ was divided into the following three subthemes with barriers and facilitators: supervision, training and education, and flexibility. The strength of evidence for these subthemes was all rated strong to very strong.

### Supervision

Multiple studies reported that supervision facilitates YPSWs in their jobs [4, 10, 16, 18, 20, 32–34, 39, 41]. Supervisors are commonly mentors from clinical staff or experienced PSWs [11, 34]. Supervision can involve coaching YPSWs on establishing boundaries, understanding and processing interactions with staff and service users, helping YPSWs understand administrative aspects and organizational language, and supporting the personal development of YPSWs [10, 15, 16, 30, 41]. Various studies reported that YPSWs often require more supervision compared to traditional clinical staff due to their young age, lack of role clarity, complex relationships with clients, and extensive use of personal lived experience [15, 30, 32, 39]. According to multiple studies, supervisors need to be influential figures, with available time and resources to offer ad hoc support to meet the supervisory needs of YPSWs [4, 15, 20, 30, 33, 41]. Moreover, studies indicate that supervision provided by experienced peer staff facilitates YPSWs to adhere to their unique values, authenticity and core objectives of peer support [30, 42].

### Training and education

Next to supervision, (ongoing) training and education can also facilitate YPSWs in practice [4, 9, 10, 15, 16, 18, 20, 32, 39, 41]. Studies reported YPSWs require skills to manage (personal) boundaries, empathize, reflect, engage and empower young people [10, 15, 16, 32]. Moreover, YPSWs also need to have an understanding of mental health disorders, conflict management and recovery principles [10, 15, 16, 32]. Some studies stressed that YPSWs require a certain level of education to perform the YPSW job well [10, 15, 16, 20]. However, another study underlines that the skills needed for on the ground implementation of YPSWs can differ significantly from the skills acquired during training [9]. Therefore, work experience opportunities prior to employment, and training delivered by staff with experiential and clinical expertise further prepares YPSWs for the job [9, 15, 41].

### Flexibility

As described in the subtheme “Role clarity”, the flexible nature of the YPSW role can be a barrier causing confusion for staff and YPSWs who need clarity and direction around the scope of the role [30, 41]. At the same time, studies report that the personal strengths and experiences brought by YPSWs requires the roles and working schedules to be tailored towards their individual needs [30, 32, 39]. Hence, a balance between flexibility and direction, whereby the job is consistently monitored and tailored towards the needs of the YPSW and the organization, is required [18, 20, 30, 32, 39, 41].

### Theme 2: *experiences of YPSWs*

The theme “experiences of YPSWs” explores how (past) experiences, personal factors and personal development of YPSWs impacts the implementation and pursuit of youth peer support services. This theme was divided into three subthemes: identity transition, control and personal factors associated with job success. The strength of evidence for these subthemes ranged from medium to very strong.

### Identity transition

Multiple studies reported that when YPSWs are first employed, they go through an identity shift from (ex-)service user to a more professional role [10, 15, 18, 20, 29, 32, 33, 37, 39–41]. Research underlined that the progression towards this new identity takes time [40, 41, 43]. Given the age and level of work experience of these young YPSWs, the readiness to fully embrace this new status as a professional can lead to YPSWs experiencing anxiety [40, 41]. Services can perceive this anxiety as a barrier to implement YPSWs. Time and space for YPSWs to explore this new identity facilitates confidence, recovery, wellbeing and readiness of YPSWs to fully embrace the role of YPSW [10, 15, 20, 32, 37, 40, 41].

### Control

The subtheme control refers to the experienced control by YPSWs in the past and present and was only mentioned in studies within the juvenile justice context [36, 37]. Working as a YPSW can empower former clients to regain control over past (adverse) experiences within the youth serving system, allowing them to rebalance their relationship with these services [36, 37]. However, when treated unequally compared to non-peer staff, YPSWs can re-experience feelings of powerlessness and lack of control [36, 37].

### Personal factors associated with job success of YPSWs

Overall, studies have found numerous personal factors that facilitate job success for YPSWs. To start, successful YPSWs often possessed adequate coping skills, resilience and the ability to bounce-back when confronted with stressful situations at work [39]. Moreover, the ability to be versatile and respond in a professional, nuanced, and authentic way in contact with young service users was also identified as an important quality for YPSWs to possess [15, 30, 33]. In terms of personal recovery, studies underline the full absence of symptoms which is not required for the YPSW role; however, YPSWs need to be capable to self-reflect and reframe recovery as understanding what happened and moving on [10, 20, 30, 39, 40]. One study found that having a supportive social network outside of work and the capacity to adhere to workplace social norms also increased acceptance, job satisfaction and credibility of YPSWs for youth mental health services [39]. In terms of age, one study acknowledged that recency of lived experience increased applicability and relevance of YPSWs to young service users [20].

### Theme 3: *Relationship between clients and YPSWs*

The theme ‘relationship between clients and YPSWs’ explores facilitators and barriers within client–YPSW relationships. This theme was divided into two subthemes: boundaries and non-judgmental attitude. The strength of evidence for these subthemes ranged from strong to very strong.

### Boundaries

Challenges surrounding personal and professional boundaries of YPSWs were an often-reported barrier [9, 10, 16, 20, 29, 30, 32, 36]. The disclosure of (past) service user status and experiences in recovery often leads to trust, genuine relationships, and equality between YPSWs and service users [9, 10, 16, 29–31, 34, 36]. However, these feelings can also lead to desires by service users to form deeper relationships with YPSWs, making it hard for YPSWs to manage personal and professional boundaries [20, 30, 32]. Therefore, YPSWs should be able to recognize and protect these subtle boundaries [30].

### Non-judgmental attitude:

An often-reported facilitator in forming a good working relationship between service users and YPSWs is the non-judgmental attitude of YPSWs [10, 18, 30, 31, 36, 40, 42]. Studies stressed that the lived experience of YPSWs creates a sense of familiarity for service users, allowing them to feel supported as they are, regardless of failure or growth [30, 31, 36, 40, 42]. Moreover, many young people have a deep sense of mistrust in youth mental health services. The non-judgmental attitude gives YPSWs an ‘outsider position’, increasing service users’ level of trust in treatment [36].

### Theme 4: *Collaboration YPSWs and non-peer staff*

The theme ‘collaboration YPSWs and non-peer staff’ was divided into the subthemes: concerns and attitudes of YPSWs and non-peer staff, co-production, and role clarity. The strength of evidence for these subthemes ranged from strong to very strong.

### Concerns and attitudes of YPSWs and non-peer staff

Studies showed several concerns and attitudes that negatively impacted the collaboration between YPSWs and non-peer staff. To start, concerns about privacy, professional boundaries and the confidentiality of service users working with YPSWs were often reported by non-peer staff [41]. Moreover, due to the perceived vulnerability and young age of YPSWs, non-peer staff were unsure about putting too much accountability on YPSWs [10, 15, 29]. As a result, YPSWs can feel belittled and ignored [39]. In terms of attitudes, a power dichotomy and professional stigma between non-peer staff and YPSWs was often reported as a barrier; both the parties feared experiential knowledge and professional practice may clash [9, 18, 20, 32, 39].

Reported facilitators to support the involvement of YPSWs included: non-peer staffs’ commitment towards involving YPSWs and creating recovery-oriented change, and joint preparation sessions to reflect on the added value of YPSWs in youth mental healthcare [10, 15, 39].

### Co-production

Various studies reported co-production as a facilitator in the collaboration process between YPSWs and non-peer staff. Co-production is described by one study as the near equal share of power between two dichotomous groups in decision-making [37]. Studies highlighted successful co-production requires YPSWs and non-peer staff to be equally engaged in the process [16, 30, 32, 33, 37, 39]. However, it has been put forward that for successful co-production or co-creation to take place, non-peer staff must recognize it takes time for some YPSWs to learn and see themselves as equal contributors [20, 37, 39]. Oftentimes YPSWs are new to the job, and they need guidance of non-peer staff [33]. Therefore, it has been suggested that non-peer staff continually invite YPSWs to communicate their ideas and feedback for the program [18, 30, 33].

### Role clarity

An often-reported barrier within the collaboration process between YPSWs and non-peer staff was a lack of understanding of YPSW roles. For YPSWs, this can lead to difficulties in setting role related boundaries, stress, burnout and job dissatisfaction [9, 10, 15, 30, 32, 33, 36, 39, 41]. YPSW roles can be diverse and should be flexible to fit the needs of the organization [20, 41]. However, this flexible and diverse nature of YPSW roles, blurs perceptions on the added value and goals of youth peer support, and creates inherit tension for YPSWs to navigate [15, 20, 30, 38, 41]. As a result, YPSWs can be insecure on how to approach their jobs, and in relationships with staff and clients, YPSWs can be mistaken for clients [10, 15, 20, 30, 41].

Studies reported that in order to facilitate understanding of YPSW roles, it is important to have numerous team members with a clear vision of the tasks the YPSW will take on [10, 41]. At the same time, YPSWs themselves must be able to educate non-peer staff about their added value [20, 30]. Studies stressed it is essential not to introduce YPSWs directly into teams without preparing the workforce on all levels of the organization [10, 16, 39]. Preparing the workforce and having dedicated advocates for youth peer support within the organization results in an improved understanding of YPSW roles [10, 16, 20, 39, 41].

### Theme 5: *organizational readiness*

The theme ‘organizational readiness’ explores the aspects required within organizations to support the implementation and pursuit of youth peer support work. Three subthemes were identified within this overarching theme: organizational requirements, training non-peer staff, and added value for the organization as a whole. The strength of evidence for these subthemes ranged from strong to very strong.

### Organizational requirements

To allow for the implementation and pursuit of youth peer support work, the organization, its culture and all people working within it need to place high value on the expertise of people with lived experience [32, 38, 39]. Various studies reported that the implementation of YPSWs requires robust planning and structuring on all levels of the organization to facilitate improved services for young people, benefit for the team as a whole, and the wellness of YPSWs [18, 29, 36, 38, 39]. Moreover, in terms of financial and contractual needs, most studies agreed that organizations need to provide adequate financing to allow for the employment of YPSWs [9, 15, 16, 18, 32, 33, 37, 41, 43]. Zero-hour contracts and voluntary positions can be a barrier for YPSWs to fully commit to the function [9, 32]. Acceptable financing of YPSWs ensures YPSWs are treated as full team members and can provide YPSWs within the justice system with an alternative opportunity to making money instead of offending [20, 32].

### Training non-peer staff

A number of studies agree that non-peer staff training facilitates the integration of YPSWs in treatment teams and organizations [10, 15, 16, 41]. Staff training results in a better understanding of the YPSW roles and gives non-peer staff a supported space to ventilate their concerns and hopes for youth peer support [10]. Successful training also provides non-peer staff with an understanding how they can utilize their personal lived experience to complement and support YPSWs [10, 15].

### Added value YPSWs for the organization as a whole

Various studies underlined that when successfully implemented, YPSWs have the ability to change the atmosphere of the service to become more youth-friendly and drive forward recovery-oriented change [10, 18, 29, 30, 36, 38, 39, 41]. This shift from a traditional medical model to a recovery-oriented system with YPSWs can improve training of clinicians, refine choices of care for young service users, and result in system wide policy changes [9]. Moreover, involving YPSWs in services can decrease relapse rates and improve the quality of life of young service users [9, 10, 41, 43], which can consequently result in more (cost)-effective care [9, 10, 41, 43].

## Discussion

This systematic review aimed to identify what we know so far about YPSW roles in treatment settings, and the barriers and facilitators for implementing and pursuing the employment of YPSWs in practice. Overall, the diversity of roles, barriers and facilitators highlight that the implementation and pursuit of youth peer support services is a multifaceted operation that requires careful planning. The variety of youth mental health services included in this review underline YPSWs can be a valuable addition to numerous youth treatment contexts, including: child and adolescent psychiatry services, community services for youth with mental health problems, youth offending services, and community services for homeless youth. This is further underlined by the numerous roles YPSWs can take on to add value to the support of young people with mild to severe mental health problems. The inclusion of YPSWs in youth mental health services can be effective in reducing disparities; YPSWs promote a diverse and inclusive workforce that is more representative of the young service users seeking help [4, 17]. YPSWs are valid role models for marginalized young people, as YPSWs commonly have lived experience being marginalized themselves [4, 17]. This is a large contrast to traditional clinical roles, requiring many years of expensive tertiary education, which is unattainable for many marginalized young people. The section below discusses the most commonly reported roles, barriers and facilitators in light of previous research.

In all roles, YPSWs actively utilize their age and lived experience to support the development and delivery of high-quality youth mental healthcare. The six roles outlined in this review describe the diverse perspectives and positions in which YPSWs act, with each role requiring different competences. The roles with the greatest strength of evidence in our review were the engagement role, the emotional support role, and the navigating and planning role. The findings for the engagement role and emotional support role are consistent with previous studies on peer support in adult mental health services. In both youth and adult mental health services (Y)PSWs promote hope, improve self-esteem, and facilitate treatment engagement of service users [4, 11, 14, 16, 31, 36]. However, the young age of YPSWs means they often have additional impacts in the engagement role and emotional support role, impacts such as the ability to engage young people through facilitating youth friendly atmospheres and the ability to provide developmentally appropriate support [4, 16, 20, 29–31, 36, 38, 40]. Within the navigating and planning role, YPSWs have the unique ability to facilitate young persons in their transition to adult mental health services [10, 31]. This role for YPSWs seems unique to youth mental health services.

Barriers and facilitators within the subtheme’s role clarity, supervision, and identity transition were most often reported and appear to be interrelated. Role clarity seems crucial for a successful implementation of YPSWs in practice [9, 15]. Echoing prior work on adult PSWs, our review pointed that a lack of role clarity results in a difficulty to maintain role related boundaries and leads to blurred perceptions of youth peer support by non-peer staff and service users [9, 15, 20, 30, 39, 44]. Our review pointed that supervision by mentors from (peer) staff facilitates YPSWs in establishing clear role boundaries [10, 39]. Besides, in agreement with previous research on adult PSWs, our review stressed that supervision facilitates the personal and professional development of YPSWs, and supports YPSWs in processing and understanding complex interactions and relationships with service users and (non-peer) staff [16, 44]. Finally, multiple studies in our review stressed that mental health services need to pay attention to the identity transition that many YPSWs experience. The young age and shift from (ex-) service user to a more professional status can be an anxiety inducing time for YPSWs [20, 41]. Time and resources to guide and supervise newly hired YPSWs facilitates the readiness of YPSWs to fully embrace their newly achieved professional status [20, 41].

The subtheme ‘Concerns and attitudes of YPSWs and non-peer staff’ warrants further discussion. Our review addressed that professional stigma and power dichotomy constitutes a barrier in the collaboration process between YPSWs and non-peer staff [9, 18, 20, 32, 39]. In agreement with a recent narrative review by Mirbahaeddin and Chreim, the power dichotomy and professional stigma of non-peer staff is influenced by the underlying dominance of medical model culture that prevails in numerous mental health services [45]. Within the medical model culture, a hierarchical structure exists whereby having a clinical or medical background is favored, and treatment is highly protocolized [45]. This medical model culture opposes the core principles of peer support based on experiential expertise, empowerment of service users, the self-determination philosophy and recovery [45, 46]. These opposing values form a barrier to the implementation of YPSW roles in organizations [45]. While training of YPSWs and non-peer staff by trainers with clinical expertise and experiential expertise facilitates the implementation of YPSWs in practice [9, 15, 41], a deeper understanding on how experiential expertise can be legitimized next to clinical expertise is required [45, 47].

### Recommendations for employing YPSWs in practice

Overall, the results on the barriers and facilitators of this review allow for several practical recommendations to implement YPSWs in practice. We discuss these recommendations in depth below.

### Recommendation 1: Set clear and realistic expectations for YPSWs

First, to fully benefit from the (experiential) expertise and authenticity of YPSWs, we advise services to set clear and realistic expectations for YPSWs. The barriers, facilitators and numerous YPSW roles outlined in this review demonstrate that the level of experience and skills required for delivering youth peer support work is high [10, 15, 16, 32]. Given the young age of YPSWs and lack of standardization of youth peer worker credentials, these high expectations may be unrealistic and can lead to exclusion of potentially successful YPSWs [16]. Allow for a certain level of flexibility in guidelines to meet the (personal) needs of YPSWs and services [18].

Future research should investigate the extent to which YPSW roles can be professionalized while simultaneously protecting the authenticity of YPSWs. Professionalization of YPSWs roles ensures more clarity and direction around the expectations for YPSWs, and allows YPSWs to be legitimatized in services favoring a traditional medical model [47]. However, professionalization of YPSW roles can also push YPSWs into more generic ways of working. With these generic ways of working comes the risk of YPSWs losing their authenticity [48].

### Recommendation 2: consider potential power imbalances in the collaboration process between YPSWs and (non)-peer staff

Second, when implementing YPSWs into existing youth mental health services, consider potential power imbalances in the collaboration process between YPSWs and other (non-peer) staff. YPSWs and (non-peer) staff both utilize different sets of expertise (experiential expertise versus clinical expertise). To ensure a successful collaboration between YPSWs and other (non-peer) staff, all staff must place equal value on both clinical expertise and experiential expertise [15, 30]. Team preparation sessions, training of YPSWs, and training of (non-peer) staff can allow for a successful collaboration between YPSWs and other (non-peer) staff [15].

While adequate financial compensation for YPSWs also ensures they are treated as full team members [20], the core principles of fostering mutuality and the ability of YPSWs to address power imbalances are compromised. When YPSWs are employed as full team members, they have to comply with demands and expectations of their employees, this puts pressure on their role and the ability for peer support to occur [47]. Practice would benefit from deeper insights into the factors influencing the collaboration process between YPSWs and (non-peer) staff.

### Recommendation 3: provide adequate time and resources to assist the personal and professional development of YPSWs

Third, we recommend services to provide adequate time and resources to assist the personal and professional development of YPSWs. YPSWs have added value for services based on their young age, developmental stage and lived experience [4, 16, 20, 29, 30, 36, 39, 40]. Due to this young age, ongoing development to adulthood, and lack of professionalization of YPSW credentials, YPSWs often require substantial guidance and training [16, 32]. Adequate time, resources and supervision on the job support YPSWs to reach their full potential [32, 39].

An extensive evaluation into which characteristics differentiate YPSWs from adult PSWs would be beneficial to create developmentally appropriate support structures and trainings for YPSWs [16]. In addition, 12 studies in our review disclosed information on whether supervision was provided by clinical staff, experienced peer staff or both. Studies indicate that receiving supervision from experienced peer staff facilitates YPSWs to adhere to their unique values and core objectives of peer support [30, 42]. A more extensive comparison of the experiences of YPSWs who were supervised by clinical staff and those supervised by experienced peer workers would provide valuable insight into the supervisory needs for YPSWs on the job. It would also provide insight in whether or not experienced peer staff are better at recognizing and addressing barriers faced by YPSWs.

### Recommendation 4: approach the implementation of YPSWs with a growth mindset

Finally, we recommend that organizations, its culture and all people working within it approach the implementation of YPSWs with a growth mindset. The implementation of YPSWs impacts all levels of the organization [10]. Once successfully implemented YPSWs can facilitate a shift from a traditional mental health system to a recovery-oriented system [9, 10]. Among others, this transition can impact staff training, treatment options and result in system wide policy changes [9]. Hence, it is crucial to embrace change and prepare all people within the organization for the change process [10].

### Strengths and limitations

Our study has several strengths. First, this study was conducted in collaboration with a YPSW specialized in research. The active role of the YPSW in screening process was beneficial for the YPSW himself; it allowed him to broaden his knowledge on the topic of youth peer support. Moreover, the advisory role of the YPSW during the data synthesis provided the review team with valuable insider perspective into youth peer support, which deepened our understanding of the results [30]. In addition, to our knowledge this is the first study that systematically reviews the globally available peer reviewed literature on youth peer support in youth mental health services. Through designing the search strategy with an information specialist, and registering the research protocol in PROSPERO, we were able to systematically identify and review the available literature on youth peer support. The qualitative and descriptive nature of our review allowed for deep insight into the nuances and process of implementing and pursuing youth peer support.

This study also comes with some limitations. To start, our review provides insight into the existing knowledge on YPSW roles in treatment settings and gives recommendations for implementing YPSWs in practice. However, as pointed by Gillard, we should be careful not to see valuable youth peer support as YPSWs that fit well within the traditional medical model commonly promoted by mental health services [47]. When we take youth peer support from the real world and mold it into existing mental health services, we risk YPSWs become another type of mental health worker there to fix the broken mind [47]. The added value of youth peer support opposes this medical model and is grounded within the authenticity of YPSWs, focusing on a strength based model and non-judgmental reciprocal relationships there to help others grow and find meaningful places within society. Thus, future research should focus on the values underpinning youth peer support, and should explore how we can support YPSWs to bring their unique set of experiential expertise and values to different practices. In line with Gillard, we as researchers need to resist the allure of the evidence base, the need to replicate YPSWs roles in para-clinical roles as the superior peer support for different settings. It is important that we gain deeper insight in what youth peer support can be in the context of existing mental health services. We must consider that the purpose and outcome measures of research should differ depending on the outcomes important to young people and YPSWs in different settings [47].

Moreover, in spite of our efforts to include a wide range of culturally diverse studies, the studies on youth peer support included in this review were all conducted in high-income westernized countries, such as: Australia, the United Kingdom, the United States, Canada, Germany, Denmark, and the Netherlands. Therefore, the cross-cultural generalizability might be limited. A deeper understanding on how youth peer support is implemented in low-income countries and different cultural contexts, is essential to capture how the core principles of peer support can be translated to various low income and culturally diverse settings [50]. Besides, it allows for more insight into the relational, psychosocial and organizational benefits of peer support [50].

In addition, our review is the first effort towards describing the existing evidence base for youth peer support for young people with a variety of mental health problems. However, the diverse array of youth serving contexts and mental health problems included within this review could have diluted some of the unique impacts YPSWs have in different settings. Besides, we were unable to find studies set in substance abuse services. This is surprising as peer support has a lengthy history in addiction treatment and has become an accepted part of treatment for substance use disorders [49]. Yet, to date studies on peer support in substance abuse services mainly concerns adults [51]. Case studies should be undertaken to provide nuance and clarity around the goals and importance of youth peer support for YPSWs and young people with diverse mental health challenges in different youth serving contexts.

Finally, our review specifically focused on YPSWs for young people with mild to severe mental health problems. Our review did not include evidence on (youth) peer support for parents and caretakers. We acknowledge that parents and caretakers can play an important role in the treatment of young people with mild to severe mental health problems [49]. Therefore, we recommend future studies to review the roles, implementation and outcomes of (Y)PSWs for parents and caretakers of young people with mild to severe mental illness.

## Conclusion

This systematic review identified the available knowledge on YPSW roles in treatment settings, and the barriers and facilitators for implementing and pursuing youth peer support in practice. Our review demonstrated that the roles of YPSWs, barriers and facilitators for implementing YPSWs seem applicable to a variety of youth mental health services. YPSWs have the ability to offer authentic and developmentally appropriate support to young people with mental and behavioral problems. To guide the involvement of YPSWs in practice, we recommend services to set realistic and clear expectations for YPSWs, consider potential power imbalances in the collaboration process between YPSWs and (non-peer) staff, provide adequate time and resources to assist the personal and professional development of YPSWs, and to approach the implementation of YPSWs with a growth mindset.

### Supplementary Information

Below is the link to the electronic supplementary material.

Supplementary file1 (DOCX 15 KB)

Supplementary file2 (DOCX 35 KB)

Supplementary file3 (DOCX 29 KB)

## Data Availability

The datasets generated and analysed during the current study are available from the corresponding author on reasonable request.
