# Correction to: A systematic review exploring youth peer support for young people with mental health problems

**DOI:** 10.1007/s00787-023-02163-2

**Published:** 2023-03-07

**Authors:** C. R. M. de Beer, L. A. Nooteboom, L. van Domburgh, M. de Vreugd, J. W. Schoones, R. R. J. M. Vermeiren

**Affiliations:** 1 LUMC Curium, Child and Adolescent Psychiatry, Leiden University Medical Center, Leiden, The Netherlands; 2 Department of Child and Adolescent Psychiatry and Psychosocial Care, Amsterdam University Medical Center, Amsterdam, The Netherlands; 3 iHUB, Rotterdam, The Netherlands; 4 Directorate of Research Policy (Formerly: Walaeus Library), Leiden University Medical Center, Leiden, The Netherlands; 5 Youz, Parnassia Psychiatric Institute, The Hague, The Netherlands

**Correction to: European Child & Adolescent Psychiatry** 10.1007/s00787-022-02120-5

The article “A systematic review exploring youth peer support for young people with mental health problems”, written by C. R. M. de Beer, L. A. Nooteboom, L. van Domburgh, M. de Vreugd, J. W. Schoones, R. R. J. M. Vermeiren was originally published electronically on the publisher’s internet portal (currently SpringerLink) on December 10, 2022 without open access. The article will be updated with open access statement as The copyright of the article changed to The Author(s) 2023 and this article is licensed under a Creative Commons Attribution 4.0 International License, which permits use, sharing, adaptation, distribution and reproduction in any medium or format, as long as you give appropriate credit to the original author(s) and the source, provide a link to the Creative Commons licence, and indicate if changes were made. The images or other third party material in this article are included in the article’s Creative Commons licence, unless indicated otherwise in a credit line to the material. If material is not included in the article’s Creative Commons licence and your intended use is not permitted by statutory regulation or exceeds the permitted use, you will need to obtain permission directly from the copyright holder. To view a copy of this licence, visit http://creativecommons.org/licenses/by/4.0/.

The original article has been corrected.

