# Supplementary material for: A systematic review exploring youth peer support for young people with mental health problems
Source: Eur Child Adolesc Psychiatry. 2022 Dec 10;33(8):2471–84. doi: 10.1007/s00787-022-02120-5 (PMC11272732; doi:10.1007/s00787-022-02120-5)
Supplement: Supplementary file 1 — Supplementary file1 (DOCX 15 KB) [file 787_2022_2120_MOESM1_ESM.docx]

**Appendix A**

*Search Strategy*

The search strategy was developed in collaboration with information specialist. The search strategy was originally developed for PsycINFO (EBSCOhost), and subsequently adapted for the other databases. Details of the complete search strategy can be obtained from the authors. See also figure 1 for a PRISMA flowchart with more information on the search strategy and included databases.

*PsycINFO Search Strategy*

Filter: Publication date 1-1-2000 to 10-6-2022

((TX("experts by experience" OR "expert by experience" OR "experience expertise" OR "experts by experienc*" OR "expert by experienc*" OR "experience expert*" OR "Patient expertis*" OR "Patients expertis*" OR "Patient's expertis*" OR "Patients' expertis*" OR "Client expertis*" OR "Clients expertis*" OR "Client's expertis*" OR "Clients' expertis*") OR TI("youth peer advocate" OR "peer helper" OR "peer specialist" OR "peer educat*" OR "peer counselor" OR "peer led" OR "peer lead*" OR "peer-to-peer" OR "peer support*" OR "peer facilitat*" OR "peer outreach" OR "peer assist*" OR "near peer" OR "peer mentor" OR "peer counseling" OR "peer counselor*" OR "PYA" OR "peer advocate*" OR "peer youth counselor") OR SU("youth peer advocate" OR "peer helper" OR "peer specialist" OR "peer educat*" OR "peer counselor" OR "peer led" OR "peer lead*" OR "peer-to-peer" OR "peer support*" OR "peer facilitat*" OR "peer outreach" OR "peer assist*" OR "near peer" OR "peer mentor" OR "peer counseling" OR "peer counselor*" OR "PYA" OR "peer advocate*" OR "peer youth counselor") OR DE(peer counseling) OR TI(("patient*" OR "client*") AND "expertis*") OR SU(("patient*" OR "client*") AND "expertis*") OR TX("formal peer support" OR "peer support worker" OR "peer support workers" OR "peer support intervention" OR "peer support interventions" OR "peer support provider" OR "peer support providers" OR "peer support program" OR "peer support program*" OR "peer support specialist" OR "peer support specialists" OR "peer support staff" OR "peer support team" OR "peer worker" OR "peer workers" OR ("peer" N3 ("worker" OR "workers")) OR (("formal support" OR "formal support*") AND ("Peer Group" OR "peer" OR "peers")))) AND (TI("Child Psychiatry" OR "Adolescent Psychiatry" OR "Psychology, Child" OR "Psychology, Adolescent" OR "Child Mental Health Services" OR "Youth Mental Health Services" OR "Adolescent Mental Health Services" OR "Child Mental Health Service" OR "Youth Mental Health Service" OR "Adolescent Mental Health Service" OR (("Mental Health Services" OR "Mental Health" OR "Mental Health" OR "Mental Disorders" OR "mental disorder*" OR "mental disease*" OR "psychiatr*" OR "psychological illness*" OR "anxiety" OR "stress" OR "Depression" OR "Behavioral Symptoms" OR "Affective Symptoms" OR "Affective Symptom" OR "Aggression" OR "Agonistic Behavior" OR "Agonistic Behaviour" OR "Bullying" OR "Burnout" OR "catatonia " OR "Compassion Fatigue" OR "Delusion" OR "Delusions" OR "Depersonalisation" OR "Depersonalization" OR "Depression" OR "Encopresis" OR "Enuresis" OR "Functional Hearing Loss" OR "functional hearing loss " OR "Malingering" OR "Mental Fatigue" OR "Obsessive Behavior" OR "Obsessive Behaviour" OR "Paranoid Behavior" OR "Paranoid Behaviour" OR "Problem Behavior" OR "Problem Behaviour" OR "Psychogenic Polydipsia" OR "Schizophrenic Language" OR "Self Mutilation" OR "Self-Injurious Behavior" OR "Self-Injurious Behaviour" OR "Stalking" OR "Suicide" OR "Wandering Behavior" OR "Wandering Behaviour" OR "Acquired Dyslexia" OR "Adjustment Disorder" OR "Adjustment Disorders" OR "Affective Disorder" OR "Affective Disorders" OR "Agoraphobia" OR "Alcohol-Related Disorder" OR "Alcohol-Related Disorders" OR "amnesia " OR "Amphetamine-Related Disorders" OR "Anorexia Nervosa" OR "Antisocial Personality Disorder" OR "Antisocial Personality Disorders" OR "Anxiety Disorder" OR "Anxiety Disorders" OR "Attention Deficit" OR "Attention Deficits" OR "autism spectrum disorder" OR "autism spectrum disorders" OR "autism" OR autistic* OR "Asperger Syndrome" OR Asperger* OR "Kanner's Syndrome" OR "Kanner Syndrome" OR "Child Development Disorders, Pervasive" OR "PDD-NOS" OR "pervasive developmental disorder not otherwise specified" OR "pervasive child development disorders" OR "Avoidant Restrictive Food Intake Disorder" OR "Binge-Eating" OR "Bipolar Disorder" OR "Bipolar Disorders" OR "Body Dysmorphic" OR "Body Dysmorphic Disorders" OR "Body Integrity Identity" OR "Body Integrity Identity Disorder" OR "Borderline Personality" OR "Borderline Personality Disorder" OR "Bulimia Nervosa" OR "Capgras Syndrome" OR "Child Behavior Disorder" OR "child behavior disorders " OR "Child Development Disorders" OR "cocaine related disorder " OR "Cognition Disorder" OR "Cognition Disorders" OR "Communication Disorder" OR "Communication Disorders" OR "Compulsive Personality Disorder" OR "Compulsive Personality Disorders" OR "Conduct Disorder" OR "Conduct Disorders" OR "Consciousness Disorder" OR "Consciousness Disorders" OR "Conversion Disorder" OR "Conversion Disorders" OR "Cyclothymic Disorder" OR "Cyclothymic Disorders" OR "Delirium" OR "Delusional Parasitosis" OR "Dementia" OR "Dependent Personality Disorder" OR "Dependent Personality Disorders" OR "Depressive Disorder" OR "Depressive Disorders" OR "Developmental Disabilities" OR "Developmental Disability" OR "Diabulimia" OR "Disruptive Behavior" OR "Disruptive Behaviour" OR "Disruptive Disorder" OR "Disruptive Disorders" OR "Dissociative Disorder" OR "Dissociative Disorders" OR "Dissociative Identity Disorder" OR "Dissociative Identity Disorders" OR "Dyspareunia" OR "Dyssomnia" OR "Dyssomnias" OR "Eating Disorder" OR "Eating Disorders" OR "Elimination Disorder" OR "Elimination Disorders" OR "Encopresis" OR "Enuresis" OR "Erectile Dysfunction" OR "Exhibitionism" OR "Factitious Disorder" OR "Factitious Disorders" OR "Feeding Disorder" OR "Feeding Disorders" OR "Fetishism" OR "Firesetting Behavior" OR "Firesetting Behaviour" OR "Food Addiction" OR "Gambling" OR "Gender Dysphoria" OR "Gender Dysphorias" OR "Histrionic Personality Disorder" OR "Histrionic Personality Disorders" OR "Hypochondria" OR "Hypochondriasis" OR "Impulse Control Disorder" OR "Impulse Control Disorders" OR "Inhalant Abuse" OR "Intellectual Disabilities" OR "Intellectual Disability" OR "Learning Disabilities" OR "Learning Disability" OR "Marijuana Abuse" OR "Marijuana Use" OR "Masochism" OR "Mood Disorder" OR "Mood Disorders" OR "Morgellons Disease" OR "Motor Disorder" OR "Motor Disorders" OR "Motor Skills Disorder" OR "Motor Skills Disorders" OR "mutism " OR "Neonatal Abstinence Syndrome" OR "Neurasthenia" OR "Neurocirculatory Asthenia" OR "Neurocognitive Disorder" OR "Neurocognitive Disorders" OR "Neurodevelopmental Disorder" OR "Neurodevelopmental Disorders" OR "Neurotic Disorder" OR "Neurotic Disorders" OR "Neurotic Disorders" OR "Night Eating Syndrome" OR "Obsessive-Compulsive Disorder" OR "Obsessive-Compulsive Disorders" OR "Paedophilia" OR "Panic Disorder" OR "Panic Disorders" OR "Paranoid Disorder" OR "Paranoid Disorders" OR "Paranoid Personality Disorder" OR "Paranoid Personality Disorders" OR "Paraphilic Disorder" OR "Paraphilic Disorders" OR "Parasomnia" OR "Parasomnias" OR "Passive-Aggressive Personality Disorder" OR "Passive-Aggressive Personality Disorders" OR "Pedophilia" OR "Personality Disorder" OR "Personality Disorders" OR "Phencyclidine Abuse" OR "Phobic Disorder" OR "Phobic Disorders" OR "Pica" OR "Premature Ejaculation" OR "Psychoses" OR "Psychosis" OR "Psychotic Disorder" OR "Psychotic Disorders" OR "reactive attachment disorder " OR "Relative Energy Deficiency in Sport" OR "Rumination Syndrome" OR "Sadism" OR "Schizoid Personality Disorder" OR "Schizoid Personality Disorders" OR "Schizophrenia" OR "Schizophrenia" OR "Schizophrenia Spectrum and Other Psychotic Disorders" OR "Schizotypal Personality Disorder" OR "schizotypal personality disorders " OR "Sexual Dysfunction" OR "Sexual Dysfunctions" OR "Sleep Wake Disorder" OR "Sleep Wake Disorders" OR "Somatoform Disorder" OR "Somatoform Disorders" OR "Stereotypic Movement Disorder" OR "Stereotypic Movement Disorders" OR "Stress Disorder" OR "stress disorders " OR "Substance Abuse" OR "Substance Withdrawal Syndrome" OR "Substance-Related Disorder" OR "Substance-Related Disorders" OR "Tic Disorder" OR "Tic Disorders" OR "Tobacco Use Disorder" OR "Tobacco Use Disorders" OR "Transvestism" OR "Trauma and Stressor Related Disorders" OR "Trichotillomania" OR "Vaginismus" OR "Voyeurism" OR "Psychiatry" OR "Psychiatric Nursing" OR "Psychology"))) OR SU("Child Psychiatry" OR "Adolescent Psychiatry" OR "Psychology, Child" OR "Psychology, Adolescent" OR "Child Mental Health Services" OR "Youth Mental Health Services" OR "Adolescent Mental Health Services" OR "Child Mental Health Service" OR "Youth Mental Health Service" OR "Adolescent Mental Health Service" OR (("Mental Health Services" OR "Mental Health" OR "Mental Health" OR "Mental Disorders" OR "mental disorder*" OR "mental disease*" OR "psychiatr*" OR "psychological illness*" OR "anxiety" OR "stress" OR "Depression" OR "Behavioral Symptoms" OR "Affective Symptoms" OR "Affective Symptom" OR "Aggression" OR "Agonistic Behavior" OR "Agonistic Behaviour" OR "Bullying" OR "Burnout" OR "catatonia " OR "Compassion Fatigue" OR "Delusion" OR "Delusions" OR "Depersonalisation" OR "Depersonalization" OR "Depression" OR "Encopresis" OR "Enuresis" OR "Functional Hearing Loss" OR "functional hearing loss " OR "Malingering" OR "Mental Fatigue" OR "Obsessive Behavior" OR "Obsessive Behaviour" OR "Paranoid Behavior" OR "Paranoid Behaviour" OR "Problem Behavior" OR "Problem Behaviour" OR "Psychogenic Polydipsia" OR "Schizophrenic Language" OR "Self Mutilation" OR "Self-Injurious Behavior" OR "Self-Injurious Behaviour" OR "Stalking" OR "Suicide" OR "Wandering Behavior" OR "Wandering Behaviour" OR "Acquired Dyslexia" OR "Adjustment Disorder" OR "Adjustment Disorders" OR "Affective Disorder" OR "Affective Disorders" OR "Agoraphobia" OR "Alcohol-Related Disorder" OR "Alcohol-Related Disorders" OR "amnesia " OR "Amphetamine-Related Disorders" OR "Anorexia Nervosa" OR "Antisocial Personality Disorder" OR "Antisocial Personality Disorders" OR "Anxiety Disorder" OR "Anxiety Disorders" OR "Attention Deficit" OR "Attention Deficits" OR "autism spectrum disorder" OR "autism spectrum disorders" OR "autism" OR autistic* OR "Asperger Syndrome" OR Asperger* OR "Kanner's Syndrome" OR "Kanner Syndrome" OR "Child Development Disorders, Pervasive" OR "PDD-NOS" OR "pervasive developmental disorder not otherwise specified" OR "pervasive child development disorders" OR "Avoidant Restrictive Food Intake Disorder" OR "Binge-Eating" OR "Bipolar Disorder" OR "Bipolar Disorders" OR "Body Dysmorphic" OR "Body Dysmorphic Disorders" OR "Body Integrity Identity" OR "Body Integrity Identity Disorder" OR "Borderline Personality" OR "Borderline Personality Disorder" OR "Bulimia Nervosa" OR "Capgras Syndrome" OR "Child Behavior Disorder" OR "child behavior disorders " OR "Child Development Disorders" OR "cocaine related disorder " OR "Cognition Disorder" OR "Cognition Disorders" OR "Communication Disorder" OR "Communication Disorders" OR "Compulsive Personality Disorder" OR "Compulsive Personality Disorders" OR "Conduct Disorder" OR "Conduct Disorders" OR "Consciousness Disorder" OR "Consciousness Disorders" OR "Conversion Disorder" OR "Conversion Disorders" OR "Cyclothymic Disorder" OR "Cyclothymic Disorders" OR "Delirium" OR "Delusional Parasitosis" OR "Dementia" OR "Dependent Personality Disorder" OR "Dependent Personality Disorders" OR "Depressive Disorder" OR "Depressive Disorders" OR "Developmental Disabilities" OR "Developmental Disability" OR "Diabulimia" OR "Disruptive Behavior" OR "Disruptive Behaviour" OR "Disruptive Disorder" OR "Disruptive Disorders" OR "Dissociative Disorder" OR "Dissociative Disorders" OR "Dissociative Identity Disorder" OR "Dissociative Identity Disorders" OR "Dyspareunia" OR "Dyssomnia" OR "Dyssomnias" OR "Eating Disorder" OR "Eating Disorders" OR "Elimination Disorder" OR "Elimination Disorders" OR "Encopresis" OR "Enuresis" OR "Erectile Dysfunction" OR "Exhibitionism" OR "Factitious Disorder" OR "Factitious Disorders" OR "Feeding Disorder" OR "Feeding Disorders" OR "Fetishism" OR "Firesetting Behavior" OR "Firesetting Behaviour" OR "Food Addiction" OR "Gambling" OR "Gender Dysphoria" OR "Gender Dysphorias" OR "Histrionic Personality Disorder" OR "Histrionic Personality Disorders" OR "Hypochondria" OR "Hypochondriasis" OR "Impulse Control Disorder" OR "Impulse Control Disorders" OR "Inhalant Abuse" OR "Intellectual Disabilities" OR "Intellectual Disability" OR "Learning Disabilities" OR "Learning Disability" OR "Marijuana Abuse" OR "Marijuana Use" OR "Masochism" OR "Mood Disorder" OR "Mood Disorders" OR "Morgellons Disease" OR "Motor Disorder" OR "Motor Disorders" OR "Motor Skills Disorder" OR "Motor Skills Disorders" OR "mutism " OR "Neonatal Abstinence Syndrome" OR "Neurasthenia" OR "Neurocirculatory Asthenia" OR "Neurocognitive Disorder" OR "Neurocognitive Disorders" OR "Neurodevelopmental Disorder" OR "Neurodevelopmental Disorders" OR "Neurotic Disorder" OR "Neurotic Disorders" OR "Neurotic Disorders" OR "Night Eating Syndrome" OR "Obsessive-Compulsive Disorder" OR "Obsessive-Compulsive Disorders" OR "Paedophilia" OR "Panic Disorder" OR "Panic Disorders" OR "Paranoid Disorder" OR "Paranoid Disorders" OR "Paranoid Personality Disorder" OR "Paranoid Personality Disorders" OR "Paraphilic Disorder" OR "Paraphilic Disorders" OR "Parasomnia" OR "Parasomnias" OR "Passive-Aggressive Personality Disorder" OR "Passive-Aggressive Personality Disorders" OR "Pedophilia" OR "Personality Disorder" OR "Personality Disorders" OR "Phencyclidine Abuse" OR "Phobic Disorder" OR "Phobic Disorders" OR "Pica" OR "Premature Ejaculation" OR "Psychoses" OR "Psychosis" OR "Psychotic Disorder" OR "Psychotic Disorders" OR "reactive attachment disorder " OR "Relative Energy Deficiency in Sport" OR "Rumination Syndrome" OR "Sadism" OR "Schizoid Personality Disorder" OR "Schizoid Personality Disorders" OR "Schizophrenia" OR "Schizophrenia" OR "Schizophrenia Spectrum and Other Psychotic Disorders" OR "Schizotypal Personality Disorder" OR "schizotypal personality disorders " OR "Sexual Dysfunction" OR "Sexual Dysfunctions" OR "Sleep Wake Disorder" OR "Sleep Wake Disorders" OR "Somatoform Disorder" OR "Somatoform Disorders" OR "Stereotypic Movement Disorder" OR "Stereotypic Movement Disorders" OR "Stress Disorder" OR "stress disorders " OR "Substance Abuse" OR "Substance Withdrawal Syndrome" OR "Substance-Related Disorder" OR "Substance-Related Disorders" OR "Tic Disorder" OR "Tic Disorders" OR "Tobacco Use Disorder" OR "Tobacco Use Disorders" OR "Transvestism" OR "Trauma and Stressor Related Disorders" OR "Trichotillomania" OR "Vaginismus" OR "Voyeurism" OR "Psychiatry" OR "Psychiatric Nursing" OR "Psychology")))) AND (AG(Childhood OR School Age OR Adolescence OR "Young Adulthood") OR TI("Child" OR "child" OR "children" OR "Infant" OR "infant" OR "infants" OR "infancy" OR "newborn" OR "newborns" OR "new-born" OR "new-borns" OR "neonate" OR "neonates" OR "neonatal" OR "neo-nate" OR "neo-nates" OR "neo-natal" OR "neonatology" OR "NICU" OR "premature" OR "prematures" OR "pre-mature" OR "pre-matures" OR "preterm" OR "pre-term" OR "postnatal" OR "post-natal" OR "baby" OR "babies" OR "suckling" OR "sucklings" OR "toddler" OR "toddlers" OR "childhood" OR "schoolchild" OR "schoolchildren" OR "childcare" OR "child-care" OR "young" OR "youngster" OR "youngsters" OR "preschool" OR "pre-school" OR "kid" OR "kids" OR "boy" OR "boys" OR "girl" OR "girls" OR "Adolescent" OR "adolescent" OR "adolescents" OR "adolescence" OR "pre-adolescent" OR "pre-adolescents" OR "pre-adolescence" OR "schoolage" OR "schoolboy" OR "schoolboys" OR "schoolgirl" OR "schoolgirls" OR "pre-puber" OR "pre-pubers" OR "pre-puberty" OR "prepuber" OR "prepubers" OR "prepuberty" OR "puber" OR "pubers" OR "puberty" OR "puberal" OR "teenager" OR "teenagers" OR "teens" OR "youth" OR "youths" OR "underaged" OR "under-aged" OR "Pediatrics" OR "Pediatric" OR "Pediatrics" OR "Paediatric" OR "Paediatrics" OR "PICU" OR children* OR schoolchild* OR "infant" OR "infants" OR "infancy" OR adolesc* OR pediat* OR paediat* OR neonat* OR toddler* OR "teen" OR "teens" OR teenager* OR preteen* OR newborn* OR postneonat* OR postnatal* OR "puberty" OR preschool* OR suckling* OR "juvenile" OR "new born" OR "new borns" OR new-born* OR neo-nat* OR neonat* OR perinat* OR underag* OR "under age" OR "under aged" OR youth* OR kinder* OR pubescen* OR prepubescen* OR "prepuberty" OR "school age" OR "schoolage" OR "school ages" OR schoolage* OR "one year old" OR "two year old" OR "three year old" OR "four year old" OR "five year old" OR "six year old" OR "seven year old" OR "eight year old" OR "nine year old" OR "ten year old" OR "eleven year old" OR "twelve year old" OR "thirteen year old" OR "fourteen year old" OR "fifteen year old" OR "sixteen year old" OR "seventeen year old" OR "eighteen year old" OR "1 year old" OR "2 year old" OR "3 year old" OR "4 year old" OR "5 year old" OR "6 year old" OR "7 year old" OR "8 year old" OR "9 year old" OR "10 year old" OR "11 year old" OR "12 year old" OR "13 year old" OR "14 year old" OR "15 year old" OR "16 year old" OR "17 year old" OR "18 year old" OR "two years old" OR "three years old" OR "four years old" OR "five years old" OR "six years old" OR "seven years old" OR "eight years old" OR "nine years old" OR "ten years old" OR "eleven years old" OR "twelve years old" OR "thirteen years old" OR "fourteen years old" OR "fifteen years old" OR "sixteen years old" OR "seventeen years old" OR "eighteen years old" OR "2 years old" OR "3 years old" OR "4 years old" OR "5 years old" OR "6 years old" OR "7 years old" OR "8 years old" OR "9 years old" OR "10 years old" OR "11 years old" OR "12 years old" OR "13 years old" OR "14 years old" OR "15 years old" OR "16 years old" OR "17 years old" OR "18 years old")))
