# Supplementary material for: A systematic review exploring youth peer support for young people with mental health problems
Source: Eur Child Adolesc Psychiatry. 2022 Dec 10;33(8):2471–84. doi: 10.1007/s00787-022-02120-5 (PMC11272732; doi:10.1007/s00787-022-02120-5)
Supplement: Supplementary file 2 — Supplementary file2 (DOCX 35 KB) [file 787_2022_2120_MOESM2_ESM.docx]

**Appendix B**

*Study Characteristics*

| Reference number | Study | Design (method) | Country | Setting | Description of peer support intervention or service | Type of peer support | Position of YPSW (paid/ volunteer) | Principal problem or diagnosis | Respondents (n) | Quality^1^ |
| --- | --- | --- | --- | --- | --- | --- | --- | --- | --- | --- |
| 28 | Coulombe et al. (2020) | Analytic  (Prospective cohort study) | Canada | Community mental health setting | Adapted peer-clinician led young adult DBT program. | Individual and group peer support | No details provided. | Young people aged 16-29 (mean age 24.7 years) with suicidal ideation and symptoms of borderline personality disorder. | Young people  (n = 76) | Fair |
| 29 | Creaney (2020) | Descriptive (Interviews & literature review) | England | Youth offending service | Exploratory interviews on youth peer support with young people and non-peer clinical staff. | Not applicable | Not applicable. | Young people on court orders. | Young people aged 13 - 18 (n = 20) Non-peer clinical staff (n = 20) | Good |
| 39 | Delman & Klodnick (2017) | Descriptive  (Focus groups) | USA | Community mental health treatment settings for young people with serious mental illness | YPSWs were implemented in various roles in two community mental health settings. Job focus varied from peer bridging and peer navigating, to providing vocational support. | Individual and group peer support | YPSWs were employed and paid. | A variety of serious mental health conditions, including:  bipolar disorder, major depression, and schizophrenia. | YPSWs aged 21 -26  (n = 7)  Supervisors of YPSWs (n = 7) | Good |
| 40 | Douglas et al. (2019) | Descriptive (Interviews) | Australia | Community service for at risk young people | Community peer mentoring program for at risk young people, the program offers weekly support groups and second monthly weekend  Camps. | Group peer support | Volunteer program for YPSWs. | At risk youth with (past) trauma(s). | YPSWs aged 18 – 23  (n = 12) | Good |
| 30 | Erangey et al. (2020) | Descriptive  (Participatory research, journaling, and semi-structured interviews) | USA | Community program for homeless youth | Community- based program called Recovery Support Services (RSS) in which YPSWs engage and build relationships with young people experiencing homelessness. | Individual and group peer support | YPSWs were paid and employed | Young people aged below 25 experiencing homelessness and addiction or mental health problems. | Program manager  (n = 1)  Peer supervisor  (n = 1)  Non-peer clinician and supervisor  (n = 1)  YPSWs  (n = 2) | Good |
| 42 | Erangey et al. (2022) | Descriptive  (Participatory research, journaling, and semi-structured interviews) | USA | Community program for homeless youth | Community- based program called Recovery Support Services (RSS) in which peer support specialists engage and build relationships young people experiencing homelessness. | Individual and group peer support | YPSWs were paid and employed | Young people below the age of 25 experiencing homelessness and addiction or mental health problems. | Program manager  (n = 1)  Peer supervisor  (n = 1)  Non-peer clinician and supervisor  (n = 1)  YPSWs  (n = 2) | Good |
| 16 | Gopalan et al. (2017) | Descriptive  (Scoping review) | USA | Variety of settings in the child serving system | Studies on youth peer led, peer delivered and peer- to- peer services in the child serving system | Reviews both individual and group peer support. | Not applicable | Young people 25 years or younger with mental, behavioral and/ or emotional problems. | Published and unpublished studies on youth peer support  (n = 30) | Good |
| 31 | Hiller- Venegas et al. (2022) | Descriptive  (Focus groups) | USA | Six county funded mental health programs serving transition aged youth with serious mental illness. | Focus groups to investigate transition aged youth perception of support received by YPSWs. | Induvial and group peer support | YPSWs were employed within six programs. No details on whether they were paid or volunteered. | Young people aged 16 – 24 with serious mental illness. | Young people  (n = 24)  . | Good |
| 32 | Hodgson et al. (2019) | Descriptive  (Participatory research and interviews) | England | Community service for young people involved in offending | Employment scheme and training for YPSWs. The YPSWs helped engage, co-produce and run the service. | Individual and group peer support | Employment scheme. | Young people involved in offending. | Non-peer clinical staff  (n = 4)  YPSWs  (n = 2) | Good |
| 9 | Hopkins et al. (2020) | Descriptive  (Survey and interviews) | Australia | Clinical child and adolescent mental health service | This study follows the implementation of peer support workers. Peer support workers were part of the recovery program and had several roles, including but not limited to mentoring, providing advice, and organizing social activities. | Individual and group peer support | Paid | Young people aged  12–25 years with psychosis. | Survey:  Non-peer (clinical and management) staff: round 1 (n = 38), round 2 (n = 24).  Interviews:  Youth peer support workers, clinical staff and management staff  (n = 9) | Fair |
| 33 | Kidd et al. (2019) | Descriptive  (Cohort study, quantitative descriptive metrics, interviews and fieldnotes) | Canada | Multiagency tertiary prevention program for youth who have experienced homelessness | Peer support workers were full team members within the multidisciplinary teams. Peer support workers engaged, mentored, ran a drop-in service and facilitated social outings. | Individual and group peer support | Paid | Young people aged 18 – 26 who have experiences homelessness. | Young people  (n = 31)  Case managers  (n = 2)  Psychologist  (n= 2)  YPSWs  (n = 2) | Good |
| 34 | King and Simmons (2022) | Descriptive  (Interviews) | Australia | Tertiary youth mental health service | Groups facilitated by two clinicians and a trained YPSWs. | Group peer support | Reimbursed as volunteers at an hourly rate. | Young people aged 15 – 25 receiving care for clinically significant and complex mental health challenges. | Young people  (n = 13) | Good |
| 10 | Lambert et al. (2014) | Descriptive  (Interview, focus groups and surveys) | England | Three clinical child and adolescent mental health services | YPSWs supported young people in the process of transitioning to adult mental health services. | Individual peer support | Paid | Young people with mental illness transitioning to adult mental health services. | YPSWs  (n = 6)  Non-peer staff:  (n = not reported)  Peer support trainer  (n = not reported) | Good |
| 35 | Lauridsen et al. (2022)  [25] | Descriptive  (Interviews and survey) | Denmark | Community program for youth experiencing symptoms of anxiety and/ or depression | Group- based intervention called “Coping with anxiety and Depression” led by YPSWs. | Group peer support | volunteer | Young people aged 15 – 25 with self- assessed or previous symptoms of anxiety and/ or depression. | Young people  (n = 483) | Good |
| 36 | Lenkens et al. (2021)  [26] | Descriptive (Interviews) | The Netherlands | Variety of youth services providing peer support to young people (16 – 30) in the criminal justice system | The YPSWs worked at a variety of (volunteer) organizations that assisted young people in the criminal justice system in the Netherlands. | Individual and group peer support | Mixed, some were paid some had unpaid volunteer positions. | Young people aged 16 – 30 involved in criminal behavior. | YPSWs (n = 20) | Good |
| 37 | Mayer & McKenzie (2017)  [28] | Descriptive (Interviews) | England | Youth community mental health charity for youth with mental health problems and criminal backgrounds | YPSW were involved in co-producing the mental health charity services. | Utilizing lived experience to co-produce a mental health charity service with other healthcare professionals. | Paid position | Young people with mental health difficulties and psychosocial problems such as a criminal record. | YPSWs  (n = 5) | Good |
| 43 | Mulfinger et al. (2018)  [29] | Analytic  (Randomized controlled trial) | Germany | Three departments of child and adolescent psychiatry | “Honest, Open, Proud” is a peer-led group program that supports both inpatient and outpatient participants with disclosure decisions to reduce self-stigma. | Group peer support | No details provided | Young people aged 13 to 18 with axis-I or axis-II disorder according to ICD-10. | Young people with mental illness  (n = 91) | Good |
| 4 | Ojeda, Jones et al. (2021) | Analytic  (Evaluation of administrative data) | USA | Administrative data of the Los Angeles County Department of Mental health services and the San Diego Department of Behavioral Health Sciences on young people receiving mental healthcare | Examines whether differing roles of YPSWs impacts service utilization of young people. | Individual and group peer support | The programs included employed YPSWs - no details rearing pay. | Young people 16 – 24 with serious mental illness. | Administrative data of youth mental health programs with YPSWs.  (n = 76) | Good |
| 17 | Ojeda, Munson et al. (2021) | Analytic  (Evaluation of administrative data) | USA | Administrative from YPSWs in the Los Angeles County Department of Mental Health Services and the San Diego Department of Behavioral Health Sciences. The data examines young people receiving mental healthcare from YPSWs. | Examines if racial concordance with YPSWs increases service use by young people. | Individual and group peer support | The programs included employed YPSWs - no details rearing pay. | Young people aged 16 – 24 with serious mental illness. | Young people  (n = 6194) | Good |
| 15 | Oldknow et al. (2014) | Descriptive  (Interview, focus groups and surveys) | England | Three clinical child and adolescent mental health services | The goal of the YPSW’s was to assist young people in their transition to adult mental health services. | Individual peer support | Employed and paid as YPSWs | Young people with mental illness transitioning to adult mental health services. | Non peer staff (management)  (n = 2)  YPSWs  (N = 6)  Non peer clinical staff  (n = not reported) | Good |
| 38 | Simmons et al. (2017) | Analytic  (Case-control study) | Australia | Enhanced primary care youth mental health service | The implementation process of a combined peer work and shared decision-making intervention by YPSWs. | Individual peer support | Paid | Young people aged 12–25 years old with mental health problems. | Historical comparison group:  Young people  (n = 80)  Intervention group: Young people  (N = 149)  YPSWs  (n = 6) | Good |
| 41 | Simmons et al. (2018) | Descriptive  (Service evaluation and surveys) | Australia | Enhanced primary care youth mental health service | The implementation process of a combined peer work and shared decision-making intervention by peer support workers. | Individual peer support | Paid | Young people aged 12–25 years old with mental health problems. | YPSWs  (n = 8)  Non- peer staff:  (n = not reported) | Poor |
| 20 | Simmons et al. (2020) | Descriptive  (Focus groups) | Australia | Enhanced primary care youth mental health service | The implementation process of a combined peer work and shared decision-making intervention by YPSWs. | Individual peer support | Paid | Young people aged 12–25 years old receiving services for a variety of mental health problems. | YPSWs  (n = 8) | Good |
| 18 | Vojtila et al. (2021) | Descriptive  (Selective review of the literature, evaluation of service, and narrative of YPSWs). | Canada | Centre for Addiction and Mental Health – focus on psychosis | Focused on the experience of an expert by experience working in treatment, research, and policy. | Providing lived experience insight in research team and collaborative care team | Paid | Young people aged 16 – 29 with early psychosis. | YPSWs  (n = 1) | Fair |

^1^Quality study - based on critical appraisal (CASP checklists).
