# Supplementary material for: A systematic review exploring youth peer support for young people with mental health problems
Source: Eur Child Adolesc Psychiatry. 2022 Dec 10;33(8):2471–84. doi: 10.1007/s00787-022-02120-5 (PMC11272732; doi:10.1007/s00787-022-02120-5)
Supplement: Supplementary file 3 — Supplementary file3 (DOCX 29 KB) [file 787_2022_2120_MOESM3_ESM.docx]

**Appendix C**

*Strength of evidence subthemes*

| Main theme | Subtheme (number of studies) | Study numbers | Quality^1^ | Context | Consistency | Strength of overall evidence |
| --- | --- | --- | --- | --- | --- | --- |
| YPSW roles in treatment settings | Engagement role  (n = 21) | 4, 9, 10, 16, 17, 18, 20, 28, 29, 30, 31, 32, 33, 34, 35, 36, 37, 38, 39, 40, 41 | Good quality: 17  Fair quality: 3  Poor quality: 1 | General | Consistent | ++++  Very strong |
|  |  | + | + | + | + |  |
| YPSW roles in treatment settings | Emotional support role  (n = 17) | 9, 10, 15, 16, 18, 20, 28, 29, 30, 31, 34, 35, 36, 37, 40, 42, 43 | Good quality: 14  Fair quality: 3  Poor quality: 0 | General | Consistent | ++++  Very Strong |
|  |  | + | + | + | + |  |
| YPSW roles in treatment settings | Navigating and planning role  (n = 11) | 10, 15, 16, 18, 20, 31, 33, 36 38, 41, 42 | Good quality: 9  Fair quality: 1  Poor quality: 1 | General: | Consistent | ++++ Verry strong |
|  |  | + | + | + | + |  |
| YPSW roles in treatment settings | Advocacy role  (n = 10) | 10, 15, 16, 18, 20, 33, 34, 36, 37, 39 | Good quality: 9  Fair quality: 1  Poor quality: 0 | General | Consistent: | ++++ Very strong |
|  |  | + | + | + | + |  |
| YPSW roles in treatment settings | Research role  (n = 3) | 16, 18, 30 | Good quality: 2  Fair quality: 1  Poor quality: 0 | General | Consistent. | ++(+/-)- Medium/ strong |
|  |  | - | +/- | + | + |  |
| YPSW roles in treatment settings | Educational role  (n = 6) | 15, 16, 18, 28, 31, 36 | Good quality: 4  Fair quality: 1  Poor quality: 0 | General | Mixed | +++- Strong |
|  |  | +- | + | + | +- |  |
| Needs of YPSW’s | Supervision  (n = 12) | 4, 10, 15, 16, 18, 20, 30, 32, 33, 34, 39, 41 | Facilitators (n = 12)  Good quality: 10 Fair quality: 1 Poor quality: 1  Barriers (n = 4)  Good quality: 4 Fair quality:  Poor quality: 0 | General | Consistent | ++++  Very strong |
|  |  | + | F: + B: + | + | + |  |
| Needs of YPSW’s | Training and education (n = 10) | 4, 9, 10, 15, 16, 18, 20, 32, 39, 41 | Facilitators (n = 10) Good quality: 7  Fair quality: 2 Poor quality: 1  Barriers (n = 0)  Good quality: 0 Fair quality: 0 Poor quality: 0 | General | Consistent | +++(+-)  Strong/ very strong |
|  |  | + | F: +/- | + | + |  |
| Needs of YPSWs | Flexibility  (n = 6) | 18, 20, 30, 32, 39, 41 | Facilitators (n = 6) Good quality: 4 Fair quality: 1 Poor quality: 1  Barriers: (n = 2)  Good quality: 1 Fair quality: 0 Poor quality: 1 | General | Consistent | +++-  Strong |
|  |  | +/- | F: +/- B: +/- | + | + |  |
| Experiences of YPSWs | Identity transition  (n = 11) | 10, 15, 18, 20, 29, 32, 33, 37, 39, 40, 41 | Facilitators n =11  Good quality: 9 Fair quality: 1 Poor quality: 1  Barriers: (n=0)  Good quality: 0 Fair quality: 0  Poor quality: 0 | General | Consistent | ++++  Very strong |
|  |  | + | F: + | + | + |  |
| Experiences of YPSWs | Control  (n = 2) | 36, 37 | Facilitators (n = 2)  Good quality: 2 Fair quality: 0 Poor quality: 0  Barriers: (n = 2)  Good quality: 2 Fair quality: 0  Poor quality: 0 | Specific | Consistent | Medium  ++-- |
|  |  | - | F: + B: + | - | + |  |
| Experiences of YPSWs | Personal factors associated with job success of YPSWs  (n = 7) | 10, 15, 20, 30, 33, 39, 40 | Facilitators (n=7) Good quality: 7 Fair quality: 0 Poor quality: 0  Barriers: (n=0)  Good quality: 0 Fair quality: 0  Poor quality: 0 | General | Consistent | Strong/ very strong  +++(+/-) |
|  |  | +/- | F: + | + | + |  |
| Relationship young service users and YPSWs | Boundaries  (n = 10) | 9, 10, 16, 20, 29, 30, 31, 32, 34, 36 | Facilitators (n = 8)  Good quality: 7 Fair quality: 1 Poor quality:0  Barriers: (n = 8)  Good quality: 7  Fair quality: 1 Poor quality:0 | General | Consistent | ++++  Very strong |
|  |  | + | F: + B: + | + | + |  |
| Relationship youth and YPSW’s | Non- judgmental  (n = 7) | 10, 18, 30, 31, 36, 40, 42 | Facilitators (n = 7)  Good quality: 6 Fair quality: 1  Poor quality:0  Barriers: (n = 0)  Good quality: 0 Fair quality: 0  Poor quality: 0 | General | Consistent | +++(+/-) Strong/ very strong |
|  |  | +- | F:+ | + | + |  |
| Collaboration YPSW’s and clinicians | Concerns and attitudes  (n = 9) | 9, 10, 15, 18, 20, 29, 32, 39, 41 | Facilitators (n = 3) Good quality: 3 Fair quality: 0 Poor quality: 0  Barriers: (n=9)  Good quality: 6 Fair quality: 2 Poor quality: 1 | General | Consistent | +++(+/-) Strong/ very strong |
|  |  | +/ - | F: + B: +/- | + | + |  |
| Collaboration YPSW’s and clinicians | Co-production  (n = 8) | 16, 18, 20, 30, 32, 33, 37, 39 | Facilitators (n = 7)  Good quality: 6 Fair quality: 1 Poor quality: 0  Barriers: (n = 4)  Good quality: 4 Fair quality: 0  Poor quality: 0 | General | Consistent | +++(+/-) Strong/ very strong |
|  |  | +/- | F: + B: + | + | + |  |
| Collaboration YPSW’s and clinicians | Role clarity  (n = 12) | 9, 10, 15, 16, 20, 30, 32, 33, 36, 38, 39, 41 | Facilitators (n = 6)  Good quality: 5 Fair quality:0  Poor quality: 1  Barriers: (n = 11)  Good quality: 9 Fair quality: 1 Poor quality: 1 | General | Mixed | +++(+/-)  Strong/ very strong |
|  |  | + | F: +/- B: + | + | +/- |  |
| Organization | Organizational requirements  (n = 14) | 9, 15, 16, 18, 20, 29, 32, 33, 36, 37, 38, 39, 41, 43 | Facilitators (n =13) Good quality: 10 Fair quality: 2 Poor quality:1  Barriers (n = 2)  Good quality: 1 Fair quality: 1 Poor quality: 0 | General | Mixed | +++- Strong |
|  |  | + | F: +/- B: +/- | + | +- |  |
| Organizational readiness | Training non-peer staff  (n = 4) | 10, 15, 16, 41 | Facilitators (n = 4) Good quality: 3 Fair quality: 0 Poor quality: 1  Barriers: (n = 0)  Good quality: 0 Fair quality: 0  Poor quality: 0 | General | Consistent | Strong +++- |
|  |  | - | F: + | + | + |  |
| Organizational readiness | Added value of YPSWs for organization as a whole  (n = 10) | 9, 10, 18, 29, 30, 36, 38, 39, 41, 43 | Facilitators (n = 10) Good quality: 7 Fair quality: 2 Poor quality: 1  Barriers: (n = 0)  Good quality: 0 Fair quality: 0  Poor quality: 0 | General | Consistent | Strong/ very strong  +++(+/-) |
|  |  | + | F: +/- | + | + |  |

^1^ Quality individual study level - based on critical appraisal (CASP checklists).
